# A Mechanism for Value-Sensitive Decision-Making

**DOI:** 10.1371/journal.pone.0073216

**Published:** 2013-09-02

**Authors:** Darren Pais, Patrick M. Hogan, Thomas Schlegel, Nigel R. Franks, Naomi E. Leonard, James A. R. Marshall

**Affiliations:** 1 Department of Mechanical and Aerospace Engineering, Princeton University, Princeton, New Jersey, United States of America; 2 Department of Computer Science and Kroto Research Institute, University of Sheffield, Sheffield, South Yorkshire, United Kingdom; 3 School of Biological Sciences, University of Bristol, Bristol, Bristol, United Kingdom; University of Maribor, Slovenia

## Abstract

We present a dynamical systems analysis of a decision-making mechanism inspired by collective choice in house-hunting honeybee swarms, revealing the crucial role of cross-inhibitory ‘stop-signalling’ in improving the decision-making capabilities. We show that strength of cross-inhibition is a decision-parameter influencing how decisions depend both on the difference in value and on the mean value of the alternatives; this is in contrast to many previous mechanistic models of decision-making, which are typically sensitive to decision accuracy rather than the value of the option chosen. The strength of cross-inhibition determines when deadlock over similarly valued alternatives is maintained or broken, as a function of the mean value; thus, changes in cross-inhibition strength allow adaptive time-dependent decision-making strategies. Cross-inhibition also tunes the minimum difference between alternatives required for reliable discrimination, in a manner similar to Weber's law of just-noticeable difference. Finally, cross-inhibition tunes the speed-accuracy trade-off realised when differences in the values of the alternatives are sufficiently large to matter. We propose that the model, and the significant role of the values of the alternatives, may describe other decision-making systems, including intracellular regulatory circuits, and simple neural circuits, and may provide guidance in the design of decision-making algorithms for artificial systems, particularly those functioning without centralised control.

## Introduction

Animals constantly make decisions, yet decision-making mechanisms and their evolution are still poorly understood in many cases. Recent years have seen a convergence of several research fields aiming to improve our understanding of general decision-making principles. Behavioural ecologists have argued for the need to combine the traditional study of animal behaviour through the lens of optimality arguments [1], with an increased understanding of the mechanisms underlying behaviour and their evolution [2]. At the same time psychologists and neuroscientists, who focus on understanding the mechanistic bases of behaviour, are increasingly focussing attention on how these mechanisms can implement optimal behaviour (*e.g.* [3]-[5]). Behavioural ecologists in the burgeoning subfield of collective animal behaviour are also interested in mechanisms, in terms of interaction rules and patterns, that generate sophisticated group decisions [6].

Some researchers have noted the parallels between these apparently disparate fields, by observing that the interaction patterns of neurons in brain circuits and animals in groups appear to be very similar [7]-[10], and also that tools and concepts from psychology and neuroscience may usefully be imported into the study of collective animal behaviour [11], [12]. These ideas have been made concrete in modelling studies where, for example, optimality analyses from neuroscience [9] or decision-making tests from psychology [8] have been applied to models of collective decision-making by social insect colonies of ants and honeybees, and in experimental studies where the parallels have successfully guided the search for decision-making mechanisms in honeybees [13], [14].

In this paper we present a comprehensive analysis of our previous empirically-motivated model of decision-making by house-hunting honeybees swarms [13], and argue that its decision-making properties may in turn guide the study of decision-making systems at other levels of biological complexity, up to individual brains, and down to intracellular decision-making circuits, as well as inform the design of artificial, decentralized decision-making systems. Our previous analysis showed that the particular pattern of `stop-signalling' observed in swarms allows them to adaptively avoid deadlock by choosing randomly when presented with two potential nest sites of equal quality, and to converge on choosing the best of two potential nest sites when there is a sufficiently large difference in their quality [13], [14].

Here, we show further aspects of value-sensitive decision-making that arise from cross-inhibitory stop-signalling. We analyse a model whose decision-dynamics are characterised by fast attraction to a one-dimensional decision manifold, followed by slower time-evolution along this manifold. We leverage a time-scale separation to reveal how the strength of cross-inhibition critically determines the decision-system response to both the difference in value and the mean value of the two alternatives. These analytic results considerably extend our previous initial analysis of this model's decision dynamics [13].

We show that stronger cross-inhibition yields a greater minimum difference in value required for discrimination between the alternatives. When the difference in value is below this minimum, the alternatives are treated as equal or nearly equal, and the cross-inhibition determines whether or not the alternatives are of sufficiently high value to warrant breaking decision deadlock. A stronger cross-inhibition increases the minimum mean value of the alternatives above which a decision deadlock is broken and the system randomly chooses one of the alternatives. When the (nearly) equal alternatives have mean value below the minimum mean value threshold, deadlock is maintained, allowing for the arrival of information on other, possibly more valuable, alternatives.

We show that cross-inhibition strength determines the minimum detectable difference in the value of alternatives, as a function of their mean value, in a manner similar to Weber's law as arising from psychological studies. We further show that for decisions over alternatives that do differ sufficiently in quality, that the stochastic decision dynamics exhibit a speed-accuracy trade-off in decision-making that depends critically on the difference in value and mean value of the alternatives, with dependence controlled by the strength of the cross-inhibition. The speed-accuracy trade-off is qualitatively similar to the statistically-optimal trade-off of the drift-diffusion model of decision-making, although we present evidence that decision-making does not achieve optimality under the parameterisations we consider here.

## Model

The decision-making model we study is an extension of our previous empirically-motivated deterministic model [13] to include stochastic fluctuations in the relevant recruitment and interaction rates. Although we shall initially describe the model in terms of house-hunting honeybees, the formulation is general and could describe any decision-maker in which accumulators compete to reach a decision threshold, are activated, decay, and inhibit each other according to the values of the alternatives they represent. For the simplest case of a decision over two alternatives, the time-evolution of the general model is described entirely by a two-dimensional system of coupled stochastic ordinary differential equations as 
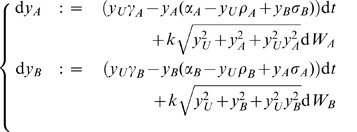
(1)where 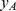 and 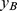 are the proportion of scout bees recruiting to potential nest sites 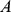 and 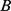 respectively, and 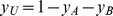 is the proportion of uncommitted scouts in the colony. Since 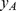 and 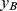 represent accumulated commitment to the alternatives, in general we refer to them as *accumulators* as is typical in theoretical neuroscience, for example [15]. Greek letters are used to denote parameters of the colony's decision-making system, that could be tuned by evolution. Latin letters are used to denote parameters of the decision problem faced by the colony that are outside of its control. Here, 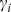 is the rate at which scouts independently discover and begin recruiting to potential nest site 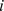, 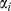 is the rate at which scouts spontaneously abandon their commitment to site 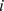, 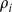 is the rate at which scouts committed to site 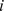 recruit uncommitted scouts via the `waggle dance' [16], and 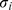 is the rate at which scouts committed to site 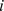 convert scouts recruiting for the competitor site to a state of non-commitment, using the `stop-signal' to disrupt waggle-dancing bees [13], [17]. Our previous experimental work has shown that this signal is delivered in a targeted manner, in that bees committed to a particular site deliver stop-signals primarily to bees dancing for competitor sites [13].

A collective decision is reached when one of the scout populations reaches a (variable) quorum threshold 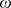. We assume that all of the rates depend on the value 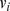 of the relevant potential nest site. As in previous work we set 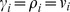 and 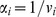 [13]. Moving beyond the model of [13], we further assume that these crucial decision rates 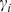, 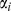 and 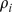 are subject to some stochastic variability, due to the inherently noisy evaluations of nest site quality 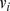 undertaken by individual scout bees; since our earlier work [13] showed that stop-signal strength should be independent of nest-site value, and since we are interested primarily in how sensory noise is processed by the decision-making system, no noise is added to rate 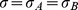. We assume independent white-noise (Wiener) processes added to the value-dependent rates, with identical variances 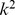. As described in Text S1 (section S.2) independent Wiener processes can be combined into a single noise term with larger variance. This is captured in the 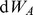 and 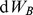 terms in Eq. 1 in which 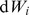 is a normally-distributed increment of the Wiener process 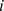, with mean 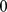 and variance 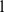. Thus the parameter 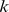 controls the noisiness, or difficulty, of the decision problem, where higher 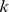 means noisier evaluations. This approach to capturing sensory noise in an infinite-population model is standard in theoretical neuroscience (*e.g.* [4]) and has previously been used to model collective behaviour of social insects (*e.g.* [9]). Note that noise captured in the Wiener processes of Eq. 1 is thus sensory noise, rather than intrinsic noise arising from finite populations of scout honeybees; correct derivation of intrinsic noise requires approaches based on the Master equation (*e.g.* [18]) and is beyond the scope of the present paper. For our dynamical systems analyses, we will set 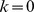 in Eq. 1, recovering the noise-free dynamics of [13], while for our stochastic decision dynamics analyses, we will set 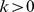.

## Results

### General Decision Dynamics — Separation of Timescales

**Figure 1:**
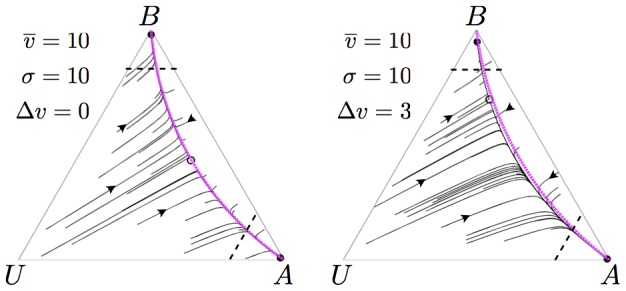
Decision-making dynamics on the unit simplex with vertex 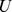 corresponding to a fully uncommitted decision-maker (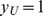), vertex 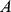 to a decision-maker fully committed to alternative 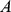 (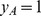, and vertex )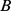 to a decision-maker fully committed to alternative 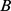 (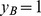). When the accumulator for alternative 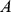 or 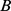 (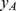 or 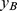) surpasses a decision threshold, illustrated with a dashed line, the corresponding alternative is selected by the decision-maker. Flow lines indicate sample noise-free trajectories over time, demonstrating fast convergence to a slow, invariant manifold. A singular perturbation analysis (Text S1) proves this separation of timescales, and gives the expression Eq. 2 for the slow manifold (magenta line), which is independent of 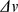 (thus, the slow manifold is the same in the right and left plots). The dynamics on the slow manifold depend on parameters of the decision problem 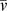 and 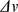 and of the cross-inhibition rate 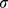; stable attractors (filled circles) can co-exist with unstable saddle-nodes (hollow circles) on the slow manifold. Thus, decision-making can be reduced to a single decision-variable; this is the form of several classic models of decision-making, including those implementing provably optimal statistical tests.

Here we present analytic results on the general decision dynamics of the model. A well-established technique for studying models of binary decision-making similar to that described in Eq. 1 is to reduce the system of equations to a one-dimensional description of the decision dynamics (*e.g.* [4], [9]). Denote the mean value of alternatives 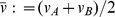 and the difference in value of alternatives 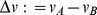. For large 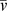 and small 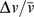, it can be shown that there is a separation of timescales; a singular perturbation analysis of the zero-noise (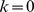) dynamics (Text S1, Figure S1) reveals fast convergence, dominated by the dynamics of the uncommitted population 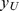, to a stable one-dimensional decision manifold, followed by slow time-evolution, dominated by the relative dynamics of the accumulators 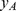 and 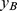, along this manifold as illustrated in Figure 1. We note that the slow manifold, defined implicitly by (Text S1) 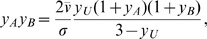
(2)


depends on 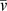 and 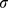 but not on 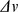, whereas the dynamics along the slow manifold depend explicitly on 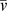, 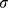, and 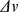 (Text S1). The analytically-calculated slow manifold is superimposed on the simulated decision-making dynamics in Figure 1 and in Figure S2, where it can be seen that the slow manifold approximates the slow dynamics well over a range of parameter values, deteriorating only when 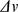 is on the order of 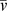.

Thus, analysing the stochastic decision dynamics along the stable one-dimensional manifold will give a good understanding of the decision-making properties of the system as a whole. This is particularly relevant because the reduced dynamics resemble classical models of binary decision-making. For example, the general one-dimensional stochastic differential equation 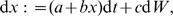
(3)


where 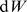 is the Wiener increment as in Eq. 1, includes Orstein-Uhlenbeck processes (OU — 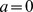, 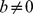) and the drift-diffusion model (DDM — 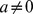, 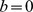) as special cases. In these models as applied to decision-making, 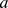 may in certain cases correspond to the signal in the stimulus presented to the decision-maker, and 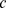 the noise in that stimulus. The decision-variable 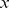 models the tendency to choose one of two alternatives where a decision is made in favor of one alternative when 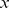 crosses a positive threshold, and the other alternative when 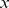 crosses a negative threshold. In the statistically-optimal DDM parameterisation, 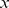 represents the log likelihood ratio of the alternatives so that 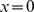 corresponds to equal evidence for each alternative.

Bogacz *et al.* previously recovered O-U processes and the DDM from two-dimensional connectionist models of choice in the visual cortex, while we recovered the DDM from two-dimensional models of nest-site selection by social insect colonies [9]. The DDM [19] is of particular interest to researchers studying decision-making because it corresponds to the statistically-optimal test for compromising between speed and accuracy of decision-making, and gives the best fits to reaction-time and error-rate distributions of subjects undertaking psychophysical decision tasks [4]. The analyses of [4] and [9] were facilitated by studying equations that converged to a linear stable manifold, whereas the stable manifold for Eq. 1 is clearly non-linear (Figure 1; Text S1). Nevertheless approximations to this manifold, as well as stochastic simulations, will enable us to analyse decision-making along it.

### Minimum Value of Acceptable Equal Alternatives

**Figure 2:**
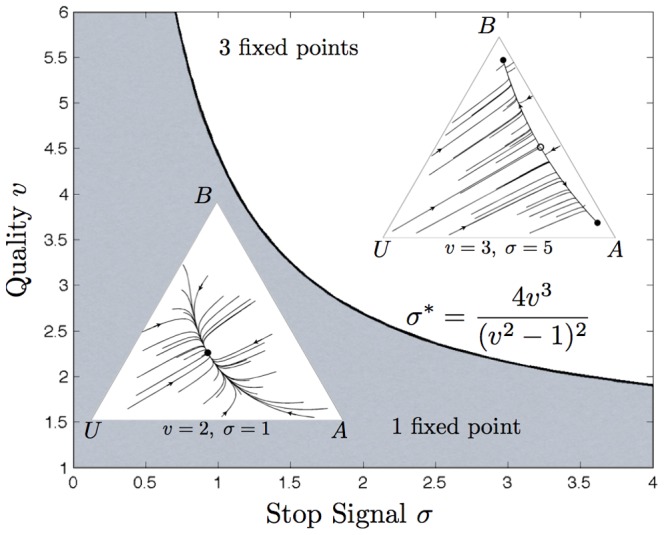
Value-dependent decision-making over equal alternatives. A critical cross-inhibition level 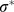 can be calculated, below which stable decision-deadlock results due to a single stable attractor on the 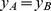 line. Increasing the strength of cross-inhibition above the critical threshold 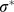, this attractor becomes unstable and two stable attractors, one for each alternative, emerge from it and rapidly move apart [13]; in this situation one alternative will thus be chosen at random by the system. As the equation and plot for 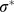 make clear, the level of cross-inhibition required to break deadlock decreases with increasing value 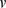 of the two alternatives. Thus, holding cross-inhibition level constant, decisions over equal but low value alternatives can result in deadlock, while decisions over equal but high value alternatives can result in a random choice. This can lead to sophisticated decision dynamics (Figs. 3 and S3).

Our previous analysis showed that the decision-making model of Eq. 1 with 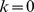, when alternatives are of equal value (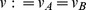), exhibits a pitchfork bifurcation as a function of increasing cross-inhibition rate 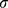 and value 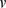 [13]. In the pre-bifurcation case, a single attractor exists at which each accumulator is of equal size, whereas in the post-bifurcation case this attractor becomes an unstable saddle point, and attractors corresponding to each alternative emerge. That is, there is a critical level of cross-inhibition 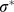 below which the decision-maker remains deadlocked between the two equal alternatives, but above which it converges to choosing one alternative at random. This threshold, plotted in Figure 2, was previously [13] calculated as 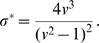
(4)


**Figure 3:**
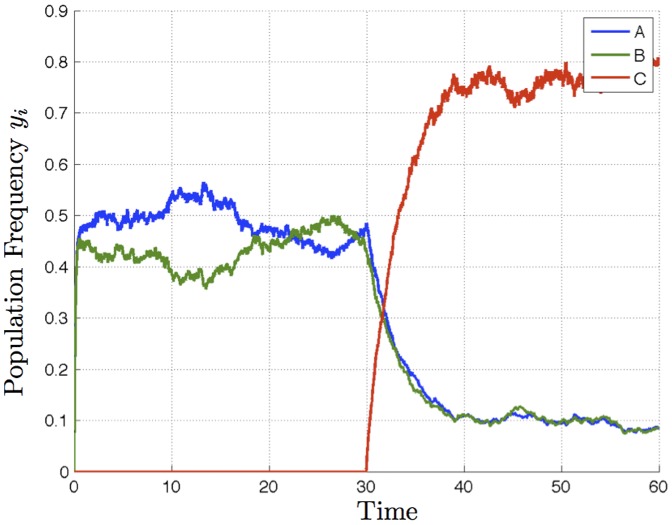
Stochastic simulation shows that for two sufficiently poor but equal alternatives, deadlock between the two persists until a third, superior alternative is discovered (at time 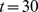), at which point it is selected by the decision-maker. The three-alternative model simulated here is a simple extension of the two-alternative model of Eq. 1, as described in section S.2 of Text S1. Noise parameter 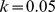.

Figure 2 demonstrates a further very useful decision-making property, that of value sensitivity. To illustrate the general principle, consider the particular case of a honeybee swarm that has discovered two equally poor potential nest sites. If both of these alternatives are of such low value to the swarm, through having insufficient volume to allow for effective colony growth and reproduction in the future, for example, then the swarm would be better off waiting to see if its scouts can discover other, higher value, alternatives in the vicinity. Figure 2 shows that, if the value of the alternatives 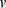 is sufficiently low given the swarm's rate of cross-inhibition 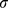 then this is precisely what happens; the recruiter populations for the two alternatives 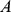 and 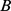 become deadlocked at equal commitment, while leaving a proportion of the swarm in the uncommitted state 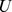 and thus available to discover alternatives through independent exploration of the environment (Figure 2; bottom-left inset). Figure 3 presents stochastic simulations of a scenario illustrating this behaviour (see Text S1), in which two equal but poor quality alternatives are discovered, and stable deadlock persists between them until a third superior alternative is discovered and subsequently chosen. This late selection of an alternative differs qualitatively from earlier models [20], in which no mechanism for adaptive deadlock was considered; in [20] a lower recruitment rate for a poor alternative gives enough time for a late-discovered good alternative to overtake the poor and reach the decision threshold first. Although we have not presented them, our model with a single-discovered alternative, in which no cross-inhibition would occur, would exhibit similar dynamics. There is experimental evidence, however, that for honeybee swarms even with only two alternatives available for discovery, times-to-discovery relative to time-to-decision are sufficient to ensure that both alternatives are discovered and a competition between them occurs [13]. The results of our model also agree qualitatively with experimental evidence that honeybee swarms are able to choose a good-quality nest site over four other medium-quality nest sites [21], which presumably requires an adaptive deadlock to be maintained between discovered medium-quality sites, until discovery of the good-quality site enables its selection.

If, however, for the same rate of cross-inhibition 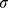 the value of the equal alternatives is sufficiently high, then the dynamics bifurcate so that the decision-maker converges on choosing one of the two alternatives at random (Figure 2; top-right inset). This illustrates a very sophisticated decision-making strategy; if information about only two alternatives is available but neither is very valuable then waiting to see if a better alternative is discovered could be sensible, whereas if the two alternatives are both of sufficient quality then quickly choosing one at random rather than wasting further time waiting for alternatives would be appropriate. Evolution could tune the level of cross-inhibition 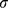 in a decision-maker to set the acceptance threshold for the value 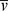 of equal alternatives to an appropriate level, given the needs of an organism and the quality of alternatives typically available in an environment, as Figure 2 illustrates.

The preceding analysis assumes an evolutionarily hard-wired level of cross-inhibition, but further sophistication is possible if one considers what might happen to our hypothetical decision-maker, considering two equal but low value alternatives, if it waits too long. Any decision-maker has finite time and resources available to make decisions; in the case of a honeybee swarm members have finite energy reserves, since they load up with honey before swarming and do not resume foraging until the swarm has found a suitable nest site [10]. If after a long period of time the swarm still only has information about the two low-value alternatives then it is reasonable to assume that no better alternatives are available as they would likely have been discovered and, in any case, the resources of the swarm are being rapidly depleted. In this scenario it would be better for the swarm to choose one of the low value nest sites than none at all. This can be achieved by progressively increasing the cross-inhibition rate 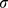; as Figure 2 indicates, by doing so a point is reached at which the value of the alternatives 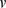, which previously resulted in stable deadlock between them, is suddenly sufficient to precipitate a random choice between the two. A stochastic simulation illustrates this process in Figure S3.

### Minimum Relevant Differences Between Equal Alternatives

**Figure 4:**
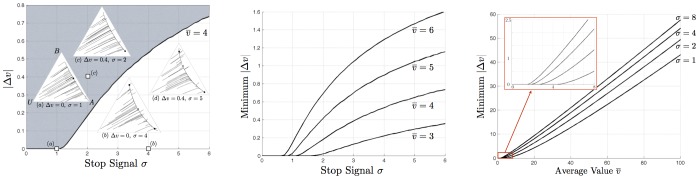
Dependence of decision-making over alternatives on absolute difference in value of alternatives 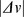, cross-inhibition strength 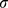, and mean value of alternatives 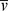. (Left) Bifurcation set as a function of 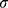 and 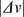, for fixed 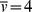. This generalises the result of Figure 2, for which 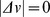. The grey region corresponds to parameters where the decision dynamics have a single stable attractor (pre-bifurcation), whereas the white region corresponds to those having two stable attractors and one saddle node (post-bifurcation). Sample phase-portraits illustrate how the positions of these fixed points change according to 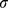 and 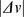. Plots (a) and (b) illustrate the results of Figure 2, in which 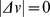. Increasing 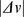 moves the stable attractor towards the superior alternative in the pre-bifurcation case (see plot (c)), although it may still correspond to a population state in which threshold is reached for neither alternative; whereas increasing 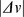 in the post-bifurcation case moves the saddle point towards the inferior alternative, thereby increasing the basin of attraction for the superior alternative (see plot (d)). Thus for a decision with given 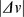 that is too low to precipitate a threshold decision, increasing 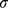 precipitates a decision, in which the more valuable alternative is more likely to be selected. (Middle) The relationship between 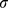 and the minimum 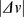 required for a unique attractor for the best alternative depends on 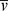. (Right) The relationship between 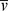 and the minimum 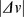 required for a single alternative to unambiguously be considered the best converges on a linear relationship, with slope determined by 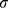. This is similar to Weber's law of just noticeable difference, observed in psychological studies, with 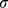 determining the Weber coefficient.

The decision dynamics of the model are sensitive not only to the value of the available alternatives but also to the absolute *difference* 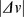 in the values of the alternatives, as illustrated in Figure 4. First, the results of Figure 2 generalize to non-zero 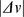; an increase in the rate of cross-inhibition 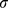 leads to a bifurcation resulting in two stable attractors, one for each alternative.

As Figure 4 (Left) shows, for small 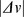 the stable deadlock point (pre-bifurcation) is moved towards the better of the two alternatives (plot (c) in 4(Left)), but may still be placed such that neither alternative reaches threshold and thereby is selected. However, for small 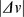, as in the case of equal value alternatives, increasing cross-inhibition ensures at least that a decision is reached; two stable attractors, one for each alternative, are introduced at the bifurcation with a saddle node between them.

For larger values of 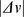, the saddle node (post-bifurcation) moves towards the inferior alternative, thereby increasing the chances that the better alternative is selected (point (d) in 4(Left)). For 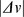 sufficiently large relative to the mean value 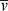 of the alternatives, the (pre-bifurcation) single stable attractor corresponding to the best alternative will be such that the decision-maker can reach the decision threshold required to select that alternative. Figure 4 (Middle) illustrates the minimum 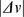 required to retain a (pre-bifurcation) single attractor for the best alternative as a function of 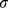 for a given 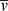.

In Figure 4 (Right) the minimum 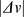 required to retain a single attractor for the best alternative is plotted as a function of 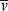. The situation in which a single attractor exists is precisely the situation in which the decision-maker could be thought of as unambiguously identifying one superior alternative from the two available, since when two attractors exists, one for each alternative, some decision trajectories lead the system towards selecting the worst of the two alternatives. Notably, the minimum 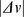 converges on a linear relationship with 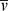, with slope determined by 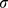 (Figure 4 (Right)). This is analogous to Weber's law of just-noticeable difference, formulated in psychology, which states that the minimum difference in stimulus intensity required to discriminate between two sources varies linearly with their mean intensity as 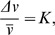
(5)


where 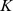 is an empirically-determined constant. From Figure 4 (Right) it is evident that 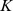 in Eq. 5 is a function of cross-inhibition rate 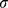. Thus cross-inhibition controls the Weber coefficient with lower rates 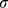 corresponding to lower Weber coefficients 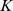, leading to a shallower increase of decision difficulty with mean value of alternatives in the decision.

### Full Dynamics Classification

**Figure 5:**
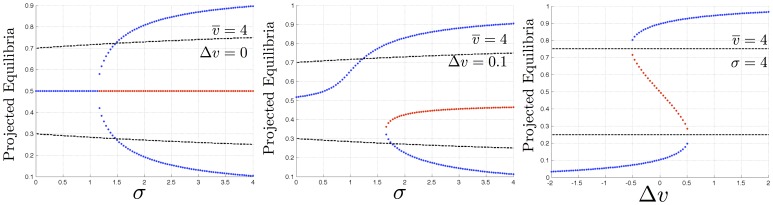
Full bifurcation behaviour of the stop-signal model of Eq. 1. According to parameterisation of the decision problem and decision-maker, the dynamics include **(i)** pitchfork bifurcation as a function of cross-inhibition rate 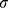 in the equal alternatives case, **(ii)** saddle-node bifurcation as a function of cross-inhibition 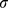 in the unequal alternatives case, and **(iii)** hysteresis as a function of difference in value of alternatives 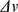. Fixed points are projected onto the 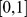 line as described in Text S1 and Figure S4. Blue dots indicate stable attractors, and red indicate unstable saddle points. Decision thresholds at 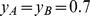 are indicated by dashed lines.

Figure 5 illustrates the full set of dynamical regimes that the stop-signal model of Eq. 1 can exhibit, as its parameters are changed. Figure 5 (Left) shows the pitchfork bifurcation with increasing cross-inhibition 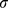 in the 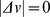 case. The dynamics in the 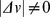 case exhibit a saddle-node bifurcation as a function of cross-inhibition rate 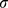 (Figure 5 (Middle)). The dynamics also exhibit a hysteretic effect as a function of difference in value of the two alternatives 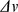 (Figure 5 (Right)). For a given value of 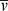, the bifurcations of the dynamics of Eq. 1, in two parameters 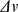 and 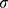, are qualitatively identical to the cusp catastrophe [22]. The plots in Figure 5 represent three slices through this cusp catastrophe bifurcation set. Each of these regimes is illustrated with stochastic simulations in the movies S1, S2, S3; the hysteresis loop implied by Figure 5 (Right) is illustrated in movie S4.

The saddle-node bifurcation of Figure 5 (Middle) clearly shows two features of the cross-inhibition rate 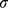. First, even for small differences in the value of alternatives relative to their mean value, increasing cross-inhibition 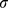 improves decision-making by moving the (pre-bifurcation) single stable attractor further and further towards the state in which there is a more highly-activated accumulator for the superior alternative. If the decision threshold, defined by dashed lines, is set to an appropriate value, increasing the cross-inhibition would therefore amplify the differences in the qualities of the the alternatives sufficiently to precipitate a decision for the better alternative, on average.

Figure 5 (Middle) also shows that too high a rate of cross-inhibition 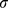 can be detrimental. If the cross-inhibition rate is increased then a stable attractor for the *inferior* alternative suddenly appears in a saddle-node bifurcation, with an unstable saddle point between it and the original stable attractor. This can be helpful to ensure a decision if a threshold is not reached pre-bifurcation; however, in the case that a threshold is reached pre-bifurcation for the superior alternative, the bifurcation might not be helpful because post-bifurcation the superior alternative is no longer a unique solution. Further increase in the cross-inhibition rate 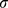 moves the inferior attractor further toward or beyond the decision threshold for the inferior alternative, and moves the saddle point closer towards equal-magnitude accumulators for each alternative (0.5 on the y-axis of Figure 5 (Middle)). Thus increasing cross-inhibition too much changes the dynamics such that there may be an increasing risk of the decision-maker converging on choosing the inferior of the two alternatives. However, as we show below higher levels of cross-inhibition can have benefits for speed-accuracy trade-offs.

In Figure 5 (Right), there is a hysteretic effect as difference in the quality of alternatives 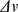 is smoothly increased and then decreased over time; this is illustrated in an animation of stochastic simulations in Text S1. While 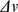 is increasing, from an initially low level, over the interval of 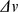 in which three fixed points co-exist (approximately −0.5 to +0.5 in the figure) the system will be in the vicinity of the lower of the two stable attractors. At a sufficiently high value of 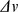 (approximately 0.5), the system will jump to the other, upper stable attractor. If 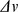 is then reduced over the same interval, the system will remain in the vicinity of the upper, stable attractor until 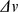 is less than approximately -0.5. While for a bee swarm, values of alternatives are unlikely to change smoothly over time in this way, this may be the case for other decision-makers, where exploitation of an alternative degrades its value, as in the example of intracellular decisions on activation of metabolic pathways considered in the Discussion. For neural decision-circuits, as also mentioned in the Discussion, laboratory experiments may be able to vary stimuli over time in this way. In both these cases the hysteretic effect of Figure 5 (Right) could act as a diagnostic that the decision-circuit used is similar in form to that described in Eq. 1.

Other authors have previously presented similar bifurcation results in different contexts for different models. For example [23] examines error rate and reaction times in connectionist models with non-linear interactions between accumulators, where these interactions serve to act as priming biases for decisions. Cell-fate decisions are analysed in [24] with respect to speed of intracellular signalling change, using the tools of bifurcation analysis. Foraging by social insect colonies, which differs from decision-making in that optimal foragers should exploit resources proportionally to their quality [25], has also been studied in this way [26], as has accuracy of collective decisions in such models [27]. While these previous studies do not, as we do, consider decisions in which a single decision-maker must choose only one option whose value they are rewarded by, they do highlight the importance of nonlinear interactions between accumulators in enabling the kinds of bifurcation behaviour presented here. In particular, nonlinear interaction between accumulators is not necessary for such behaviour; indirect nonlinear interaction, through accumulator populations competing for a finite pool of uncommitted individuals [26], for example, is sufficient.

### Speed-Accuracy Trade-offs

As noted above, several classical models of decision-making, including the DDM and the (un)stable O-U process, are described using equations of stochastic motion on a line. The separation of timescales result presented above demonstrates that the decision dynamics converge rapidly to a line, along which they slowly diffuse. Of particular interest in decision-making models are speed-accuracy trade-offs [28]-[30], and the optimal compromise between these two quantities [4], [9]. We therefore undertook preliminary numerical investigations (described in the Text S1) into the stochastic behaviour of the decision system under different parameterisations, once the system has converged to the stable decision-manifold, and until it crosses a decision-threshold.

**Figure 6:**
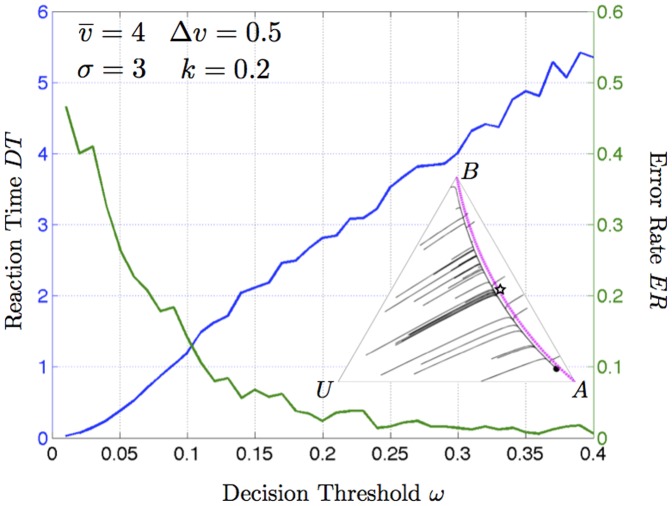
Speed-accuracy trade-off when differences between alternatives are sufficiently large that a single attractor for the best alternative exists; the observed speed-accuracy trade-off is qualitatively similar to that realised by the statistically-optimal drift-diffusion model of decision-making (see Text S1). When two attractors for alternatives of different values exist, however, the presence of the unstable saddle-node can improve error rate without compromising reaction time (see Text S1 and Figure S5).

Figure 6 presents a classic speed-accuracy trade-off, for a parameterisation that results in only a single attractor for the best alternative available. In Figure S5 in Text S1 we present numerical analyses of other cases, which highlight further interesting decision dynamics; in particular, we show for certain parameterisations that having an attractor for the incorrect alternative can actually *improve* reaction time, without compromising decision accuracy (compare top left and top right plots of Figure S5). The fact that this improvement is possible indicates that decision-making along the stable manifold with a single attractor is *not* a statistically-optimal drift-diffusion process under the parameterisations studied here.

## Discussion

Although motivated by and presented in terms of decision-making by house-hunting honeybee swarms, our model exhibits a number of beneficial decision-making qualities that we might expect other organisms to exhibit. At the heart of our analysis is the observation that, in a choice, an animal is typically rewarded by the value of the chosen alternative, rather than whether or not it chose the best. In particular the model decision-maker displays a sensitivity to the absolute as well as the relative value of the alternatives under consideration; this enables it to wait for information on better alternatives to arise when considering equally poor alternatives, but to spontaneously choose one equal alternative at random when both are good enough relative to a crucial decision-making parameter, the rate of stop-signalling, or cross-inhibition, 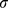. The decision-maker exhibits other properties observed in psychological studies, such as speed-accuracy trade-offs, and Weber's law of just-noticeable difference. The increasing rate of cross-inhibition may also improve the energetic costs of decision-making, although possibly at the expense of decision accuracy (as discussed in Text S1 and Figure S6). Our investigation has focussed on analytic treatment of the noise-free equations and stochastic simulations of speed-accuracy trade-offs and decision dynamics for binary decision-problems. Much work remains to be done in extending these analyses, for example to decisions over more than two alternatives.

Having suggested that our model might describe adaptive decision-making in general, what are the prospects for finding similar decision-making networks in other species? The form of the model equations is that of chemical reaction kinetics, in which interactions between chemical species are described by `mass action' terms. Therefore, there is the potential for intra-cellular regulatory networks to implement these decision-dynamics quite easily, for example in deciding for which of a number of available substances to activate the associated metabolic pathway. Evidence that single-cells can, for example, implement Bayesian-estimation through intra-cellular signalling [31], or exhibit Weber's law in gene regulatory pathways [32], [33] indicates that such decision-making at the cell level is entirely plausible. Mutual inhibition also features in models of transcription in cell-fate decisions [24].

Another obvious class of decision models that invite comparison are those developed to describe neural networks for decision-making in simple perceptual decision tasks, such as those that take place in the primate visual cortex. A variety of accumulator models have been studied for their ability to fit experimental data, as well as implement optimal decision strategies (*e.g.* [4]). Optimal parameterisation of many such models requires evidence-accumulating pathways to interact [4], which the cross-inhibition mechanism in our model also implements. While optimality analyses in these models do take account of variable rewards for correct choices (*e.g.* [34], [35]), they do not typically account for the fact that in real animals incorrect choices over, for example, food items still result in a reward, albeit one that is not the best available. Recently however, there is increasing interest in combining ideas from psychophysics, such as the Drift-Diffusion Model (DDM) [19], with the study of value integration processes (*e.g.* [36], [37]).

Many accumulator models, like the classic DDM, also struggle with the correct choice when presented with zero net evidence, such as when choosing between two stimuli of equal average magnitude, and thus cross decision thresholds only through random drift. When choice of either alternative would result in an equal reward, such behaviour is clearly sub-optimal. Proposals to deal with this include implementing `urgency signals' or collapsing decision thresholds over time [38], [39], and the use of time-dependent sensory gain, and asymmetric inhibition between evidence pathways [38]. Our model differs from these proposed mechanisms, in that it spontaneously exhibits behaviour like that of an unstable Ornstein-Uhlenbeck process in order to break deadlock, according to the value of the alternatives under consideration and the strength of cross-inhibition.

Our non-linear model differs from the linear formulation of accumulator models underlying many analyses (*e.g.* [4]). The non-linear interaction terms of our model can, however capture neural activation dynamics; the logistic activation curve for neural populations in an accumulator model, used in [23], are qualitatively similar to `activation patterns' in the stop-signalling model, and [23] derives behaviour qualitatively similar, although not identical, to the stable-deadlock and deadlock-breaking results presented above. It is not unreasonable to expect convergent evolution to arrive at the same simple solution to the problem of value-dependent decision-making, in systems as diverse as single cells, honeybee swarms, and vertebrate nervous systems.

## Supporting Information

Figure S1 **Level curves in *x,z* coordinates.**
(TIFF)

Figure S2
**Comparison between the analytically computed slow manifold *h*(*x*) plotted in magenta and simulations of the stop-signaling dynamics (S1).** The match between the analytical slow manifold and the simulations is excellent, except for the case 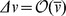, 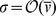. For this set of plots, 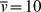, 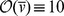 and 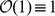.(TIFF)

Figure S3
**Simulations of the stochastic dynamics (S20) with time-varying stop-signal.** A deadlocked population is able to converge to a decision for one of two equal alternatives by slowly ramping up the stop-signal; the critical value of stop-signal for the pitchfork bifurcation is marked on the bottom plot. Noise parameter 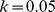.(TIFF)

Figure S4
**Illustration of equilibrium 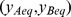 projected orthogonally onto 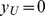.** These projected equilibria are plotted in Figure 5 of the main text.(TIFF)

Figure S5
**Increasing stop-signalling rate 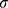 has energetic benefits, as the total number of individuals involved in decision-making at any point in time is reduced.** However, given the wisdom-of-the-crowds effect, this may have an adverse effect on collective accuracy, as fewer individual value estimates are pooled.(TIFF)

Figure S6
**Error Rate (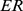, green) and Reaction Time (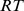, blue) for the stochastic decision-making dynamics (S20) stopsde as a function of decision threshold 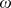.** Parameters are indicated on each plot: (a) standard parameterisation from figure 6 in main text, (b) bistability with difference in value of alternatives, resulting from stronger cross-inhibition, (c) monostability for larger difference in value of alternatives and stronger cross-inhibition, (d) symmetric bistability when alternatives are equal in value.(TIFF)

Matlab Code S1 Matlab code for stochastic simulation models.
(ZIP)

Movie S1 Locations of fixed-points, and simulated stochastic trajectories, as a function of varying stop-signal level (equal alternatives).
(MP4)

Movie S2 Locations of fixed-points, and simulated stochastic trajectories, as a function of varying stop-signal level (unequal alternatives).
(MP4)

Movie S3 Locations of fixed-points, and simulated stochastic trajectories, as a function of varying difference in quality of alternatives.
(MP4)

Movie S4 Hysteretic effect as result of smoothly varying difference in quality of intervals repeatedly over a fixed interval.
(MP4)

Text S1 Further information on analytic and simulation results.
(PDF)
