# Supplementary figures and images for: A Mechanism for Value-Sensitive Decision-Making
Source: PLoS One. 2013 Sep 2;8(9):e73216. doi: 10.1371/journal.pone.0073216 (PMC3759446; doi:10.1371/journal.pone.0073216)

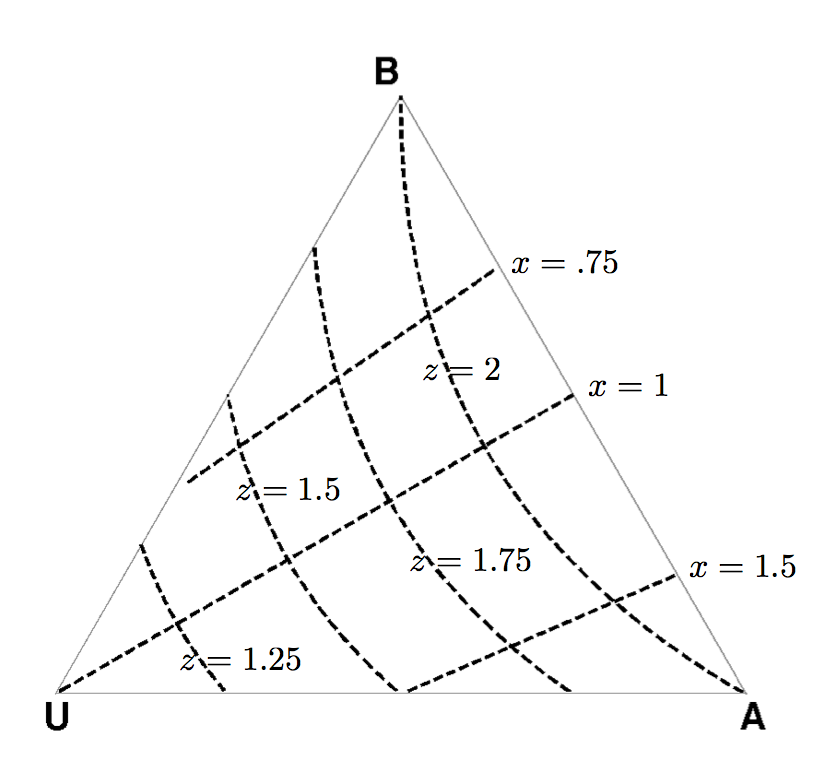

Supplement: Figure S1 — Level curves in x,z coordinates. (TIFF) [file pone.0073216.s001.tiff]

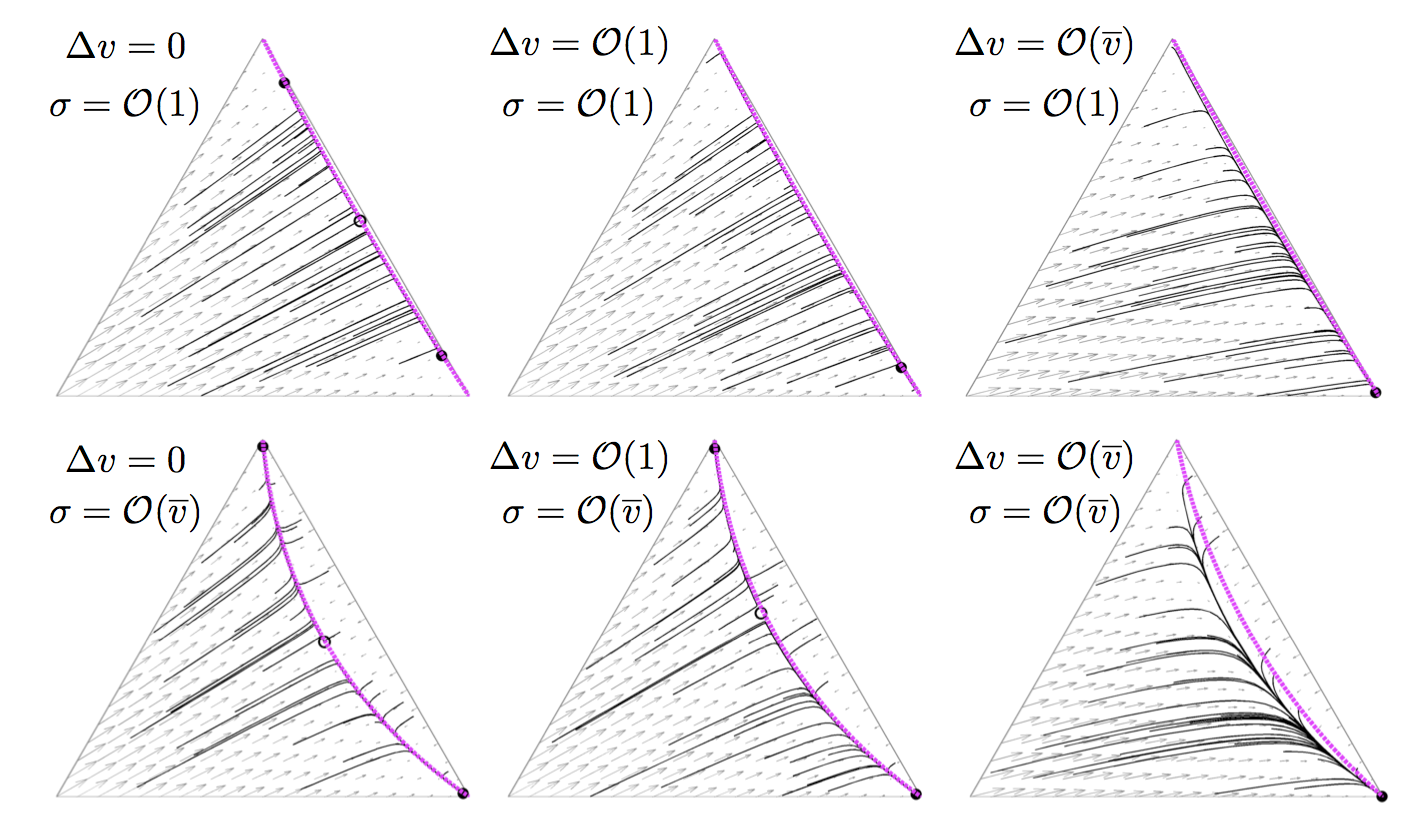

Supplement: Figure S2 — Comparison between the analytically computed slow manifold h(x) plotted in magenta and simulations of the stop-signaling dynamics (S1). The match between the analytical slow manifold and the simulations is excellent, except for the case , . For this set of plots, , and . (TIFF) [file pone.0073216.s002.tiff]

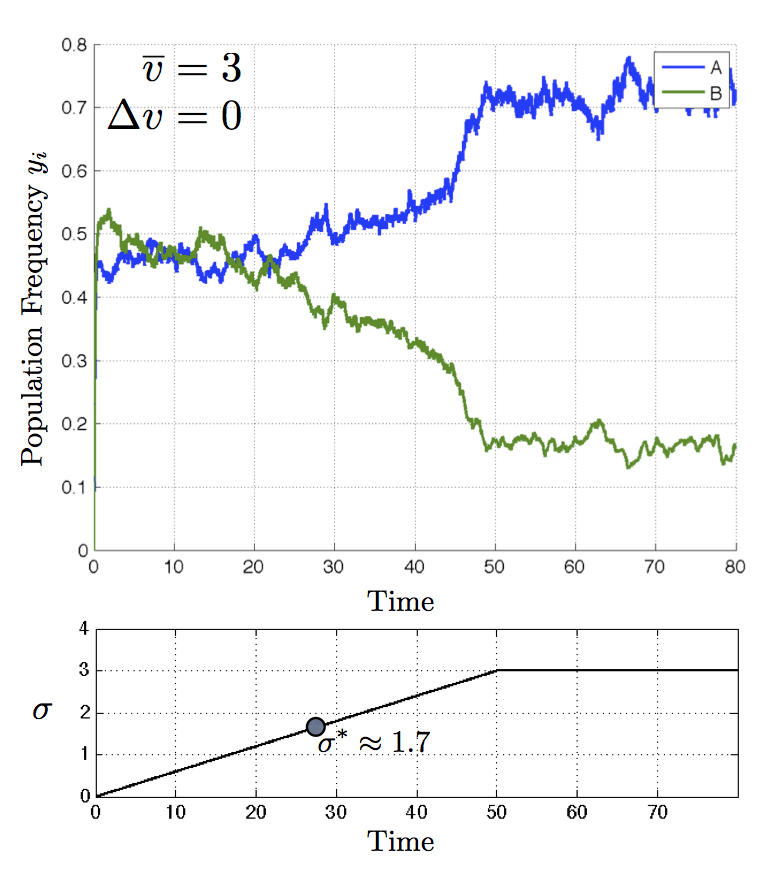

Supplement: Figure S3 — Simulations of the stochastic dynamics (S20) with time-varying stop-signal. A deadlocked population is able to converge to a decision for one of two equal alternatives by slowly ramping up the stop-signal; the critical value of stop-signal for the pitchfork bifurcation is marked on the bottom plot. Noise parameter . (TIFF) [file pone.0073216.s003.tiff]

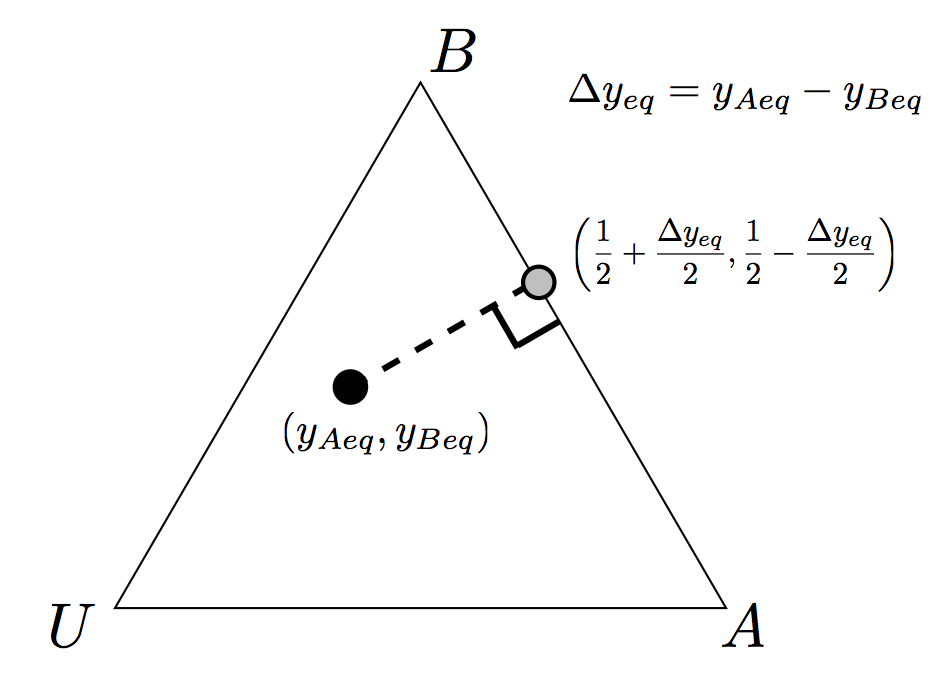

Supplement: Figure S4 — Illustration of equilibrium projected orthogonally onto . These projected equilibria are plotted in Figure 5 of the main text. (TIFF) [file pone.0073216.s004.tiff]

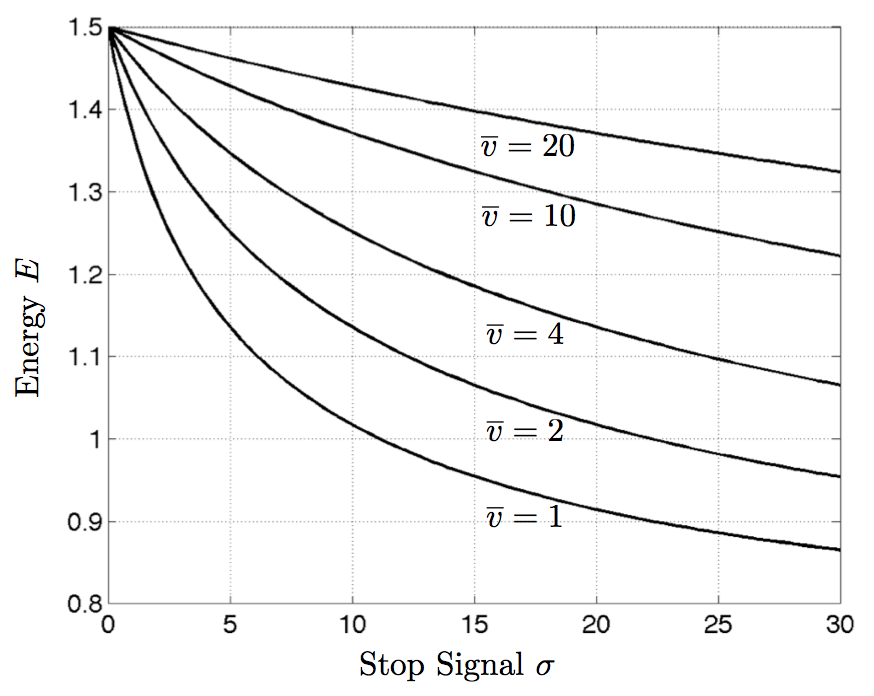

Supplement: Figure S5 — Increasing stop-signalling rate has energetic benefits, as the total number of individuals involved in decision-making at any point in time is reduced. However, given the wisdom-of-the-crowds effect, this may have an adverse effect on collective accuracy, as fewer individual value estimates are pooled. (TIFF) [file pone.0073216.s005.tiff]

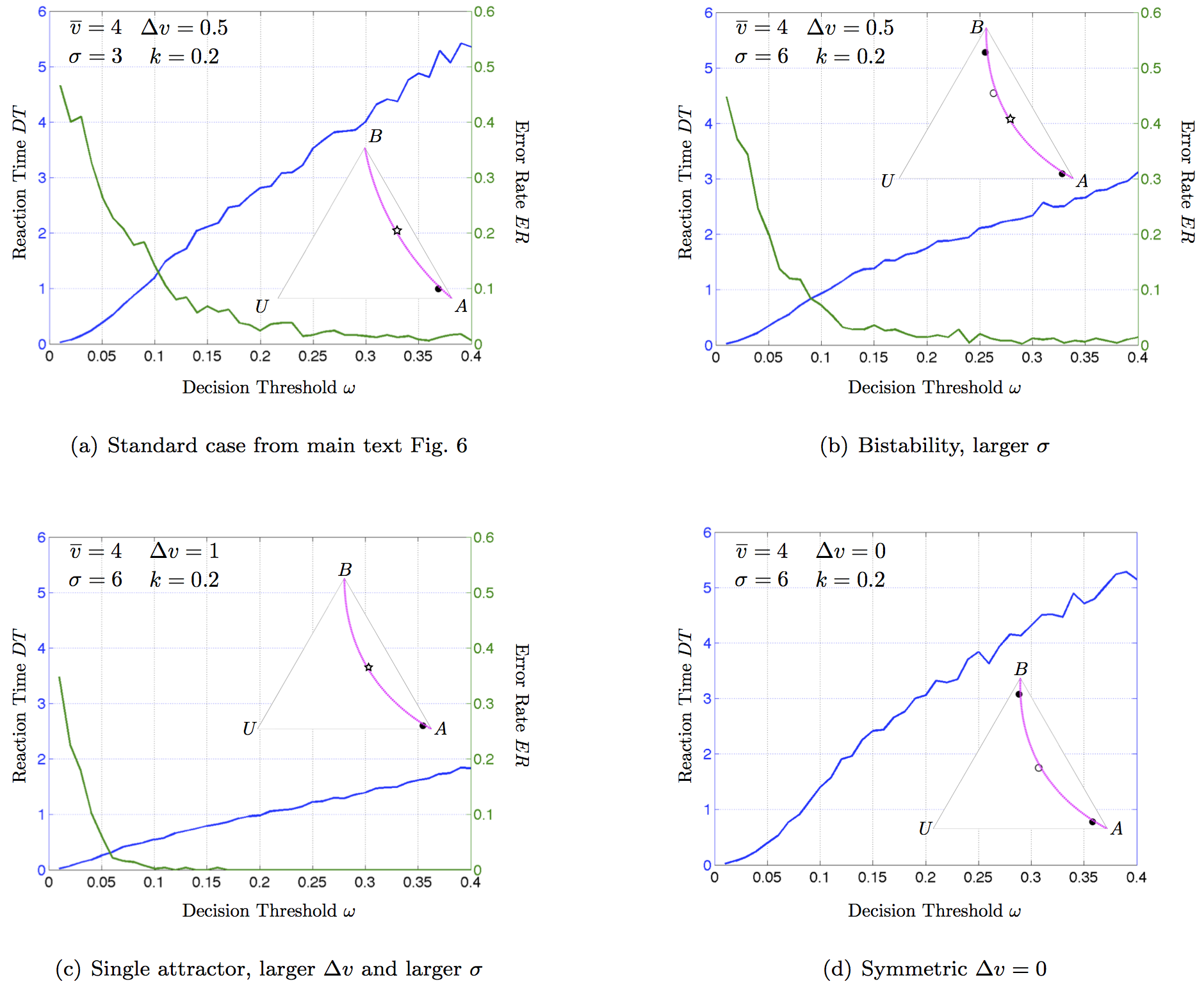

Supplement: Figure S6 — Error Rate (, green) and Reaction Time (, blue) for the stochastic decision-making dynamics (S20) stopsde as a function of decision threshold . Parameters are indicated on each plot: (a) standard parameterisation from figure 6 in main text, (b) bistability with difference in value of alternatives, resulting from stronger cross-inhibition, (c) monostability for larger difference in value of alternatives and stronger cross-inhibition, (d) symmetric bistability when alternatives are equal in value. (TIFF) [file pone.0073216.s006.tiff]

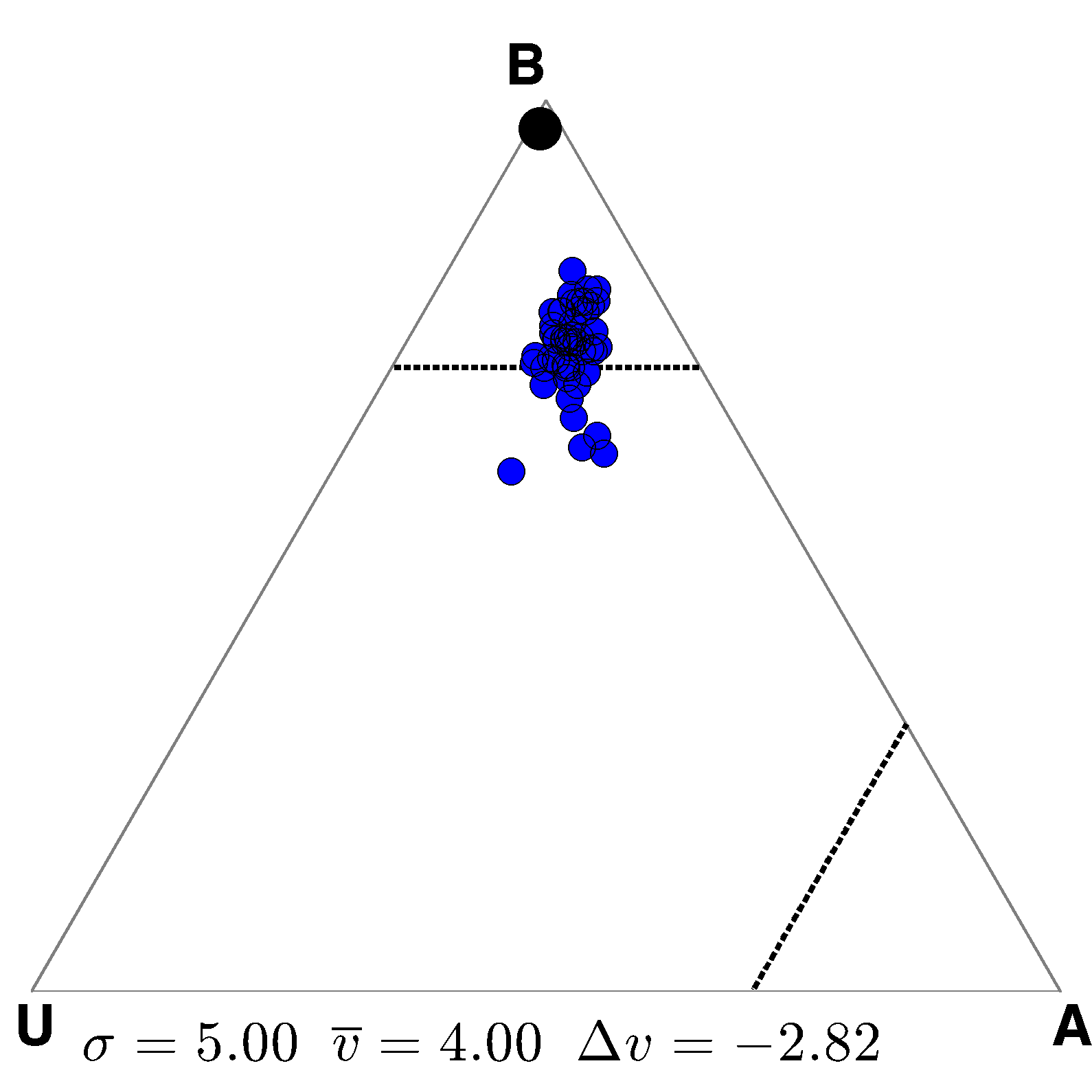

Supplement: Matlab Code S1 — Matlab code for stochastic simulation models. (ZIP) [file pone.0073216.s007.zip › Matlab SI/Stop_Stochastic/f2.png]
