# Supplementary material for: A Mechanism for Value-Sensitive Decision-Making
Source: PLoS One. 2013 Sep 2;8(9):e73216. doi: 10.1371/journal.pone.0073216 (PMC3759446; doi:10.1371/journal.pone.0073216)
Supplement: Text S1 — Further information on analytic and simulation results. (PDF) [file pone.0073216.s012.pdf]

Supplementary Information for 'A Mechanism for Value-Sensitive Decision-Making'

D. Pais, P. M. Hogan, T. Schlegel, N. R. Franks, N. E. Leonard and J. A. R. Marshall

S.1 Timescale Separation

This section comprises the details of the timescale separation calculation for the stop-signalling dynamics given by

$$\begin{aligned}\frac{dy_A}{dt} &= \frac{-2y_A}{2\overline{v}+\Delta v} + \left(\overline{v}+\frac{\Delta v}{2}\right) y_U(1+y_A) - \sigma y_A y_B \\ \frac{dy_B}{dt} &= \frac{-2y_B}{2\overline{v}-\Delta v} + \left(\overline{v}-\frac{\Delta v}{2}\right) y_U(1+y_B) - \sigma y_A y_B.\end{aligned} \tag{S1}$$

We perform a nonlinear coordinate transformation of the dynamics (S1) and apply singular perturbation theory to separate timescales and derive an analytic expression for the slow manifold (heteroclinic connections). Assuming large $\overline{v}$, define $\epsilon := \frac{1}{\overline{v}}$ as the small parameter for the timescale separation calculations. We follow the notation used in Chapter 11 of [3] and apply Tikhonov's Theorem (Theorem 11.1 in [3]) to prove the timescale separation.

Standard Singular Perturbation Model

Consider the coordinate transformation $(y_A, y_B) \mapsto (x, z)$ given by

$$\begin{aligned} z &= (1+y_A)(1+y_B) \\ x &= \frac{1+y_A}{1+y_B}. \end{aligned} \tag{S2}$$

Define the 2-simplex $\Delta_2 = \left\{(x,y) \in \mathbb{R}^2 | x \geq 0,\ y \geq 0 \text{ and } x+y=1\right\}$. The above transformation is well-defined on the domain $(y_A, y_B) \in \Delta_2$ since the Jacobian of the linearization has non-zero determinant $\frac{-2(1+y_A)}{1+y_B}$ on Δ_2. The inverse transformation is given by

$$\begin{aligned} y_A &= \sqrt{zx} - 1 \\ y_B &= \sqrt{\frac{z}{x}} - 1. \end{aligned} \tag{S3}$$

Note that $(y_A, y_B) \in \Delta_2$ implies that $z \in \left[1, \frac{9}{4}\right]$ and $x \in \left[\frac{1}{2}, 2\right]$. Level curves in x, z coordinates on the simplex are illustrated in Fig. S2.

Define the functions $\alpha(x,z)$ and $\beta(x,z)$ as

$$\alpha(x,z) = \sqrt{zx} + \sqrt{\frac{z}{x}} = 2 + y_A + y_B \tag{S4}$$

$$\beta(x,z) = \sqrt{zx} - \sqrt{\frac{z}{x}} = y_A - y_B. \tag{S5}$$

Then,

$$y_A y_B = z + 1 - \alpha,\ y_A + y_B = \alpha - 2, \text{ and } y_U = 3 - \alpha. \tag{S6}$$

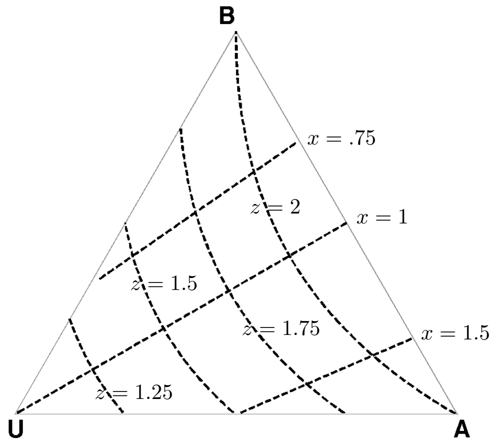

Figure S1: Level curves in x,z coordinates.

Computing the time derivative of z and substituting from (S1), (S4), (S5) and (S6) we get

$$\begin{aligned}\dot{z} &= \dot{y}_B(1+y_A) + \dot{y}_A(1+y_B) \\ &= \frac{2}{4\overline{v}^2 - \Delta v^2}\left[\Delta v\ \beta(x,z) - 2\overline{v}(2z - \alpha(x,z))\right] \\ &+ 2\overline{v}z(3-\alpha(x,z)) - \sigma\alpha(x,z)(z+1-\alpha(x,z)).\end{aligned} \tag{S7}$$

Computing the time derivative of x and substituting from (S1), (S4), (S5) and (S6) we get

$$\begin{aligned}\dot{x} &= \frac{\dot{y}_A(1+y_B) - \dot{y}_B(1+y_A)}{(1+y_B)^2} \\ &= \frac{x\Delta v(4z - 2\alpha(x,z)) - 4x\overline{v}\beta(x,z)}{4\overline{v}^2 z - z(\Delta v)^2} + x\Delta v(3-\alpha(x,z)) \\ &+ \frac{\sigma x\beta(x,z)}{z}(z+1-\alpha(x,z)).\end{aligned} \tag{S8}$$

Let $d = \sigma/\overline{v}$. The transformed dynamics (S7), (S8) can be written as a singular perturbation problem in standard form with small parameter $\epsilon = 1/\overline{v}$,

$$\begin{aligned}\epsilon\frac{dz}{dt} &= 2z(3-\alpha) - d\alpha(z+1-\alpha) \\ &+ \frac{2\epsilon^3\beta\Delta v}{4-\epsilon^2(\Delta v)^2} - \frac{4(2z-\alpha)\epsilon^2}{4-\epsilon^2(\Delta v)^2} \qquad =: g(x,z,\epsilon)\end{aligned} \tag{S9}$$

$$\begin{aligned}\frac{dx}{dt} &= x(3-\alpha)\Delta v + \frac{\sigma\beta x}{z}(z+1-\alpha) \\ &- \frac{4\beta x\epsilon}{4z - z\epsilon^2(\Delta v)^2} + \frac{x(4z-2\alpha)\epsilon^2\Delta v}{4z - z\epsilon^2(\Delta v)^2} \qquad =: f(x,z,\epsilon).\end{aligned} \tag{S10}$$

To ensure that the $\epsilon \to 0$ limit is well-defined we assume

$$\lim_{\overline{v}\to\infty}\frac{\Delta v}{\overline{v}} = \lim_{\epsilon\to 0}\epsilon\Delta v = 0. \tag{S11}$$

Slow Manifold Calculation

The slow manifold is given by the root of $g(x,z,0)=0$.

$$\begin{aligned} g(x,z,0)=0 &\implies 2z(3-\alpha(x,z)) - d\alpha(x,z)(z+1-\alpha(x,z)) = 0 \\ &\implies 2zy_U - d\alpha(x,z)(z+1-\alpha) = 0 \\ &\implies 2(1+y_A)(1+y_B)y_U = d(3-y_U)(y_Ay_B) \\ &\implies \frac{d}{2}y_Ay_B = \frac{y_U(1+y_A)(1+y_B)}{3-y_U}. \end{aligned} \tag{S12}$$

Equation (S12) is an implicit expression for the slow manifold. Define the function $\hat{x} = \sqrt{x} + \frac{1}{\sqrt{x}}$. Then $\alpha(x,z) = \sqrt{z}\hat{x}$. In order to obtain an explicit expression we rewrite (S12) in the (x,z) coordinates as follows,

$$\begin{aligned} g(x,z,0)=0 &\implies 2z(3-\alpha(x,z)) - d\alpha(x,z)(z+1-\alpha(x,z)) = 0 \\ &\implies 2z(3-\sqrt{z}\hat{x}) - d\sqrt{z}\hat{x}(z+1-\sqrt{z}\hat{x}) = 0 \\ &\implies 6\sqrt{z} - 2z\hat{x} - d\hat{x}z - d\hat{x} + d\hat{x}^2\sqrt{z} = 0 \\ &\implies (2+d)\hat{x}z - (d\hat{x}^2+6)\sqrt{z} + d\hat{x} = 0. \end{aligned} \tag{S13}$$

Equation (S13) is quadratic in $\sqrt{z}$. The solutions to the quadratic are given by

$$\sqrt{z} = \frac{d\hat{x}^2 + 6 \pm \sqrt{D}}{2(2+d)\hat{x}}, \tag{S14}$$

where the discriminant $D = d^2\hat{x}^4 + 36 + 4d\hat{x}^2 - 4d^2\hat{x}^2$. Hence we have two distinct solutions for the slow manifold given by

$$z = \left(\frac{d\hat{x}^2+6+\sqrt{D}}{2(2+d)\hat{x}}\right)^2, \left(\frac{d\hat{x}^2+6-\sqrt{D}}{2(2+d)\hat{x}}\right)^2. \tag{S15}$$

The second solution in (S15) lies outside the feasible domain $z \in [1, \frac{9}{4}]$ (and correspondingly $(y_A, y_B) \in \Delta_2$) and is hence rejected.

To summarize, the slow manifold is given by

$$\boxed{\begin{aligned} &z = \left(\frac{d\hat{x}^2+6+\sqrt{D}}{2(2+d)\hat{x}}\right)^2 =: h(x) \\ &\text{where } \hat{x} = \sqrt{x} + \frac{1}{\sqrt{x}} \text{ and } D = (d\hat{x}^2+6)^2 - 4d\hat{x}^2(2+d). \end{aligned}} \tag{S16}$$

Attractivity of the Slow Manifold

The boundary layer dynamics are given by

$$\frac{dz}{d\tau} = g(x, z+h(x), 0), \tag{S17}$$

where x is treated as fixed parameter. Stability of the boundary layer dynamics requires the exponential stability of its origin, uniformly in the fixed parameter x [3]. To test for exponential stability of the origin, we compute the Jacobian of the dynamics (S17) evaluated at the origin

$$\left.\frac{\partial}{\partial z} g(x, z+h(x), 0)\right|_{z=0} = -\frac{1}{12}\sqrt{1296 + \frac{(1+x)^2\sigma\left(24x + (x-1)^2\sigma\right)}{x^2}} \tag{S18}$$

and note that $\left.\frac{\partial}{\partial z} g(x, z+h(x), 0)\right|_{z=0} < 0$ for all $x \in [\frac{1}{2}, 2]$.

The reduced dynamics on the slow manifold defined by (S16) are given by

$$\begin{aligned} \dot{x} &= f\left(x, h(x), 0\right) \\ &= \frac{\sigma x}{h(x)}\left[h(x) + 1 - \alpha\left(x, h(x)\right)\right]\beta\left(x, h(x)\right) + x(3 - \alpha\left(x, h(x)\right)\Delta v. \end{aligned} \tag{S19}$$

Comparisons to Simulations

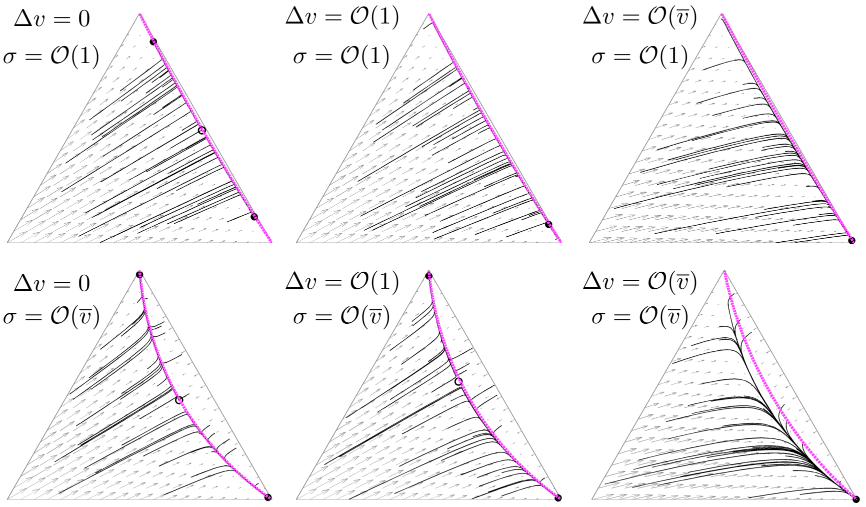

Figure S2: Comparison between the analytically computed slow manifold $h(x)$ plotted in magenta and simulations of the stop-signaling dynamics (S1). The match between the analytical slow manifold and the simulations is excellent, except for the case $\Delta v = \mathcal{O}(\overline{v})$, $\sigma = \mathcal{O}(\overline{v})$. For this set of plots, $\overline{v} = 10$, $\mathcal{O}(\overline{v}) \equiv 10$ and $\mathcal{O}(1) \equiv 1$.

In Fig. S2, we plot a comparison of the analytically derived slow manifold (S12) (or equivalently (S16)) and trajectories of the dynamics (S1) for various combinations of parameters σ and Δv, for large $\overline{v}$ (taken to be $\overline{v} = 10$ for this plot). The match between the approximation and the trajectories is excellent, except for the case when both Δv and σ are large. This case violates the limiting conditions of the singular perturbation calculation (S11).

S.2 Stochastic Simulations

The stochastic decision-making dynamics are given by

$$\begin{aligned} dy_A &= (\gamma_A y_U + \rho_A y_A y_U - \alpha_A y_A - \sigma_B y_A y_B)\, dt + k\sqrt{y_U^2 + y_A^2 + y_U^2 y_A^2}\, dW_A \\ dy_B &= (\gamma_B y_U + \rho_B y_B y_U - \alpha_B y_B - \sigma_A y_B y_A)\, dt + k\sqrt{y_U^2 + y_B^2 + y_U^2 y_B^2}\, dW_B, \end{aligned} \tag{S20}$$

for choices over two alternatives, where $y_U = 1 - y_A - y_B$, and

$$\begin{aligned} dy_A &= (\gamma_A y_U + \rho_A y_A y_U - \alpha_A y_A - \sigma_B y_A y_B - \sigma_C y_A y_C)\, dt + k\sqrt{y_U^2 + y_A^2 + y_U^2 y_A^2}\, dW_A \\ dy_B &= (\gamma_B y_U + \rho_B y_B y_U - \alpha_B y_B - \sigma_A y_A y_B - \sigma_C y_B y_C)\, dt + k\sqrt{y_U^2 + y_B^2 + y_U^2 y_B^2}\, dW_B \\ dy_C &= (\gamma_C y_U + \rho_C y_C y_U - \alpha_C y_C - \sigma_A y_A y_C - \sigma_B y_B y_C)\, dt + k\sqrt{y_U^2 + y_C^2 + y_U^2 y_C^2}\, dW_C \end{aligned} \tag{S21}$$

for choices over three alternatives, where $y_U = 1 - y_A - y_B - y_C$. The parameter k in (S20) and (S21) sets the level of stochasticity or noisiness in the decision-making process; higher values of k correspond to noisier evaluations.

To derive the noise terms of the above equations, white noise is added to each value-dependent transition rate in the original ordinary differential equations (ODEs), as motivated in the main paper, to capture sensory noise in individual valuations of alternative nest sites. As described in [1], for example, multiple independent white noise (Wiener) processes can be combined into a single Wiener process whose standard deviation is

the square root of the sum of the constituent processes' variances. Thus, for example, the rate terms for spontaneous commitment to and abandonment of a nest site, from the original noise-free ODE model, with white noise terms having standard deviation k added to these rates, can be written

$$y_U(\gamma_A dt + k dW_{\gamma_A}) - y_A(\alpha_A dt + k dW_{\alpha_A}), \tag{S22}$$

where dW_{γ_A} and dW_{α_A} are independent Wiener processes with unit variance/standard deviation, capturing normally-distributed error around, respectively, the spontaneous commitment and abandonment rates for site A, γ_A and α_A. Note that for simplicity we assume the standard deviations in the normally-distributed fluctuations around the rates are all k; relaxing this would still allow a single Wiener process to be constructed, but without a single parameter controlling the overall noisiness of the system. Since our motivation in studying the stochastic model is primarily to show that the deterministic dynamics we derived are not sensitive to noise, the former modelling approach is satisfactory.

As noted above, the sum (or difference) of two independent Wiener processes with standard deviations a and b is itself a Wiener process with standard deviation $\sqrt{a^2+b^2}$. Applying this fact to the above expression we derive a stochastic ODE with a single Wiener process

$$(y_U\gamma_A - y_A\alpha_A)dt + k\sqrt{y_U^2 + y_A^2}dW, \tag{S23}$$

where dW is a third Wiener process having unit variance, since $\sqrt{k^2y_U^2 + k^2y_A^2} = \sqrt{k^2}\sqrt{y_U^2 + y_A^2}$. The full noise terms in the above equations of the stochastic model are thus constructed in this way by adding Wiener fluctuations to all value-dependent rates in the original deterministic ODE model and summing them into a single Wiener process; note that value-dependent rates include all the rates *except* the stop-signalling rate, which previous work has shown should not be value-dependent [4].

Two illustrative simulations of the stochastic dynamics (S20) and (S21) are shown in Fig. S3 and Fig. 3 in the main text:

- Breaking symmetric deadlock (Fig. S3): The decision-making process is between two equal alternatives with value $v_A = v_B = 3$, hence the dynamics are described by (S20). The stop-signal σ ramps up linearly from an initial value of $\sigma = 0$ to a final value of $\sigma = 3$. The increasing stop-signal enables the deadlock to be broken when $\sigma > \sigma^* \approx 1.7$ with one alternative randomly winning out. This simulation is motivated in part by the fact that elapsed time might influence the computations that underlie decision processes since prolonged deliberation can be expensive [2].

- Deadlock enables better final outcome (Fig. 3, main text): For a system tuned to intermediate levels of stop-signal ($\sigma = 1$ here), alternatives of low quality result in deadlock. This enables the system to wait for other potentially better alternatives to arrive into the mix and precipitate a decision. In this simulation, a third high-quality option ($v_C = 4$) enters at $t = 30$ and dominates. The dynamics are described by (S21)

S.3 Animations Corresponding to Fig. 5

- *FPlocs1.mp4, FPlocs2.mp4, FPlocs3.mp4*: Bifurcation plot and stochastic simulations corresponding to Fig. 4 of the main text. Each snapshot of the simplex shows 50 trajectories initialized at the origin $y_A = y_B = 0$ (correspondingly $y_U = 1$), with noise parameter $k = 0.1$, parameters $\overline{v}$, Δv and σ indicated below each simplex, and for time $t \in [0, 30]$.

  Circles are equilibria (filled stable, hollow unstable) of the deterministic dynamics (S1), and the dashed lines are quorum thresholds $y_A = 0.7$ and $y_B = 0.7$. The inset bar plots show the fraction of trajectories reaching each threshold; 'none' indicates trajectories reaching neither threshold in the given time (plotted in cyan). For $\Delta v = 0$, trajectories reaching either threshold are plotted in blue. For $\Delta v \neq 0$ trajectories reaching the correct threshold (one with higher v_i) are plotted in green, those reaching the incorrect threshold are in red.

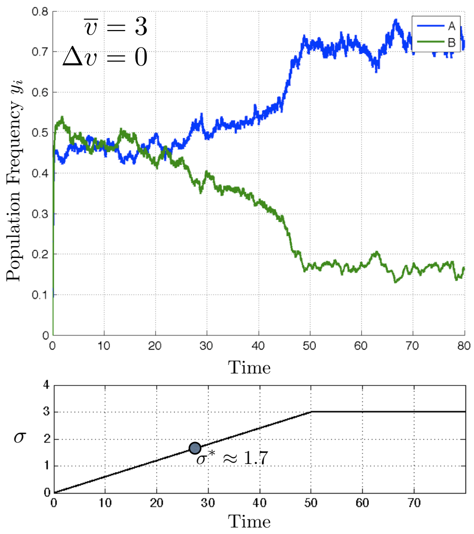

Figure S3: Simulations of the stochastic dynamics (S20) with time-varying stop-signal. A deadlocked population is able to converge to a decision for one of two equal alternatives by slowly ramping up the stop-signal; the critical value of stop-signal for the pitchfork bifurcation is marked on the bottom plot. Noise parameter $k = 0.05$.

- *hysteresis.mp4*: Cloud of points moving according to the stochastic dynamics (S20) with noise parameter $k = 0.1$ and other parameters shown on the plot. This simulation illustrates the hysteretic effect associated with continuously varying the difference between alternatives Δv for a given mean value $\overline{v}$ and stop signal σ.

S.4 Projected Equilibria

The bifurcations plots in Fig. 5 of the main text are obtained by projecting equilibria of the dynamics (S1) orthogonally onto the $y_U = 0$ line, as illustrated for an equilibrium (y_{Aeq}, y_{Beq}) in Fig. S4. For any given equilibrium (y_{Aeq}, y_{Beq}), the orthogonal projection onto the $y_U = 0$ line is given by the point $(\frac{1}{2}+\frac{\Delta y_{eq}}{2}, \frac{1}{2}-\frac{\Delta y_{eq}}{2})$ where $\Delta y_{eq} = y_{Aeq} - y_{Beq}$. The location of the point along $y_U = 0$ is given by $P = \frac{1}{2} + \frac{\Delta y_{eq}}{2}$, which is the quantity that is plotted on the vertical axis of Fig. 5.

S.5 Speed-Accuracy Trade-offs

Error Rate (ER) and Reaction Time (RT) plots for the stochastic stop-signalling dynamics (S20) are shown in Fig. S6 for four sets of parameters. For each of these sets, ER and DT are plotted as a function of the decision threshold ω. We use 50 values of ω in the range $\omega \in [0, 0.4]$ shown on the x-axis to construct each plot. In each plot, for a given value of ω, 500 simulations of the stochastic dynamics (S20) are run with initial condition at the zero evidence point ($y_A = y_B =: y_0$) on the corresponding slow manifold. The slow manifold and initial condition (shown as a star at (y_0, y_0)) are plotted in the insets. Each simulation is stopped when a trajectory reaches the threshold (either $y_A > y_0 + \omega$ or $y_B > y_0 + \omega$). RT is the mean time taken by the 500 trajectories to reach either threshold. ER is the fraction of trajectories reaching the incorrect threshold (one with lower value v_i). Each of the plots in Fig. S6 requires $500 \times 50 = 25000$ runs of the stochastic dynamics (S20).

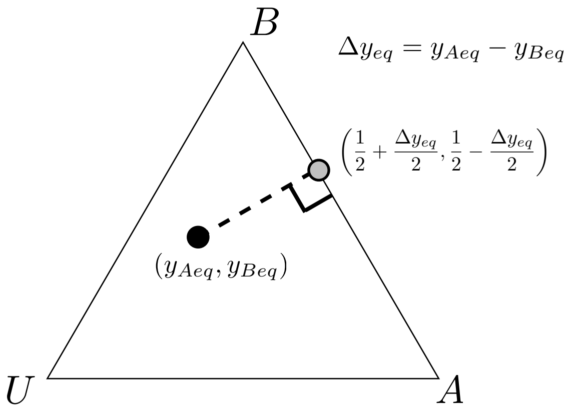

Figure S4: Illustration of equilibrium (y_{Aeq}, y_{Beq}) projected orthogonally onto $y_U = 0$. These projected equilibria are plotted in Fig. 5 of the main text.

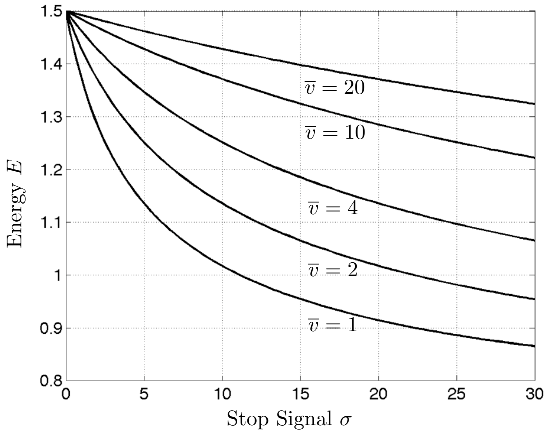

Figure S5: Increasing stop-signalling rate σ has energetic benefits, as the total number of individuals involved in decision-making at any point in time is reduced. However, given the wisdom-of-the-crowds effect, this may have an adverse effect on collective accuracy, as fewer individual value estimates are pooled.

S.6 Energetic Expenditure

Stop-signalling may also benefit the energy consumption of the swarm during decision-making. Energetic considerations are often ignored in studies of decision-making, yet are of crucial importance for decision-makers embodied in the real world. As mentioned in the main text, for example, house-hunting honeybee swarms have fixed energetic resources since members do not forage until decision-making is completed. A similar argument can be applied to decision-making in neural circuits, where activation of a neural population will consume energy over time. In Fig. S5 we show how increasing the stop-signalling rate σ improves energy consumption by the swarm by reducing the number of individuals involved in the decision-making. However, this is at the possible expense of decision accuracy, as discussed below.

Expected energetic expenditure of the swarm can be characterised as

$$\mathbb{E}(\text{energy expenditure}) \propto \int_{1/2}^{2} (y_A(x) + y_B(x))\,\text{Prob}(x)dx \tag{S24}$$

where, $x \in [1/2, 2]$ parameterizes the the one-dimensional slow manifold (S16). $y_i(x)$ is the inverse transform

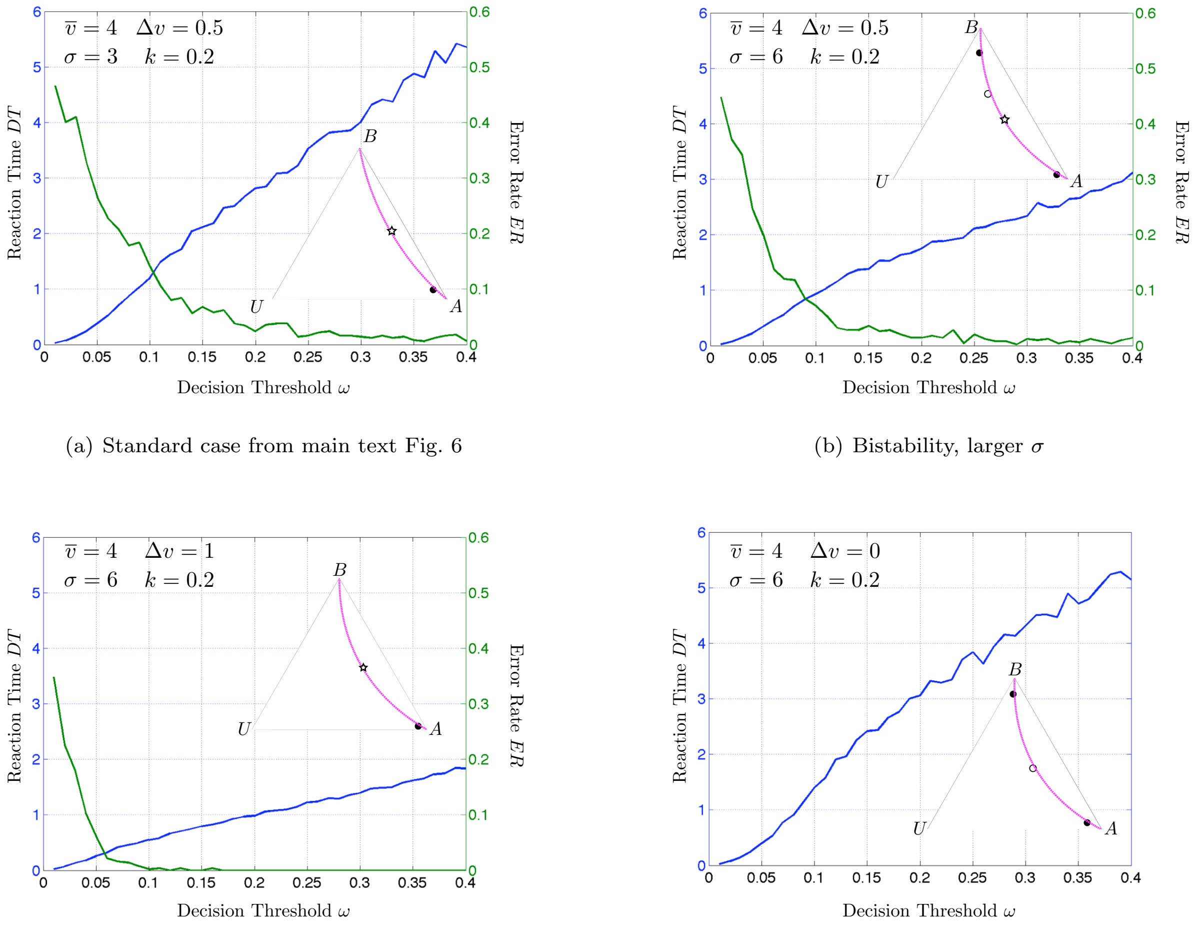

(a) Standard case from main text Fig. 6

(b) Bistability, larger σ

(c) Single attractor, larger Δv and larger σ

(d) Symmetric $\Delta v = 0$

Figure S6: Error Rate (ER, green) and Reaction Time (RT, blue) for the stochastic decision-making dynamics (S20) as a function of decision threshold ω. Parameters are indicated on each plot: (a) standard parameterisation from figure 6 in main text, (b) bistability with difference in value of alternatives, resulting from stronger cross-inhibition, (c) monostability for larger difference in value of alternatives and stronger cross-inhibition, (d) symmetric bistability when alternatives

(S3) that recovers the number of individuals committed to site i when the decision-system is at point x on the decision manifold, and Prob(x) is the probability that the decision-system is at point x on the manifold at any given point in time. For simplicity let us assume that the swarm is equally likely to occupy all of the points x on the manifold. This is a conservative assumption, since during decision-making, and before decision thresholds are reached, the system will spend more of its time at points closer to the centre of the manifold ($x = 1$, corresponding to equal commitment of scouts to the two alternatives, *i.e.* $y_A = y_B$), and it is precisely these points whose energetic cost is reduced the most by increased stop-signalling. Thus, by assuming a uniform distribution over the stable manifold we underweight the points on the manifold that have the greatest energetic saving for increased stop-signalling. Nevertheless, plotting the energetic expenditure of (S24) as a function of σ shows that increasing the rate of stop-signalling reduces the expected energetic costs of decision-making (Fig. S5).

It should be noted, however, that although reducing energetic expenditure during decision-making is beneficial, there is a disadvantage not accounted for by our continuous model. In a decision-making system with discrete decision-components, whether they be honeybee scouts, or members of neuronal populations, reduc-

ing the number of components participating in a process will increase the noise associated with the process. By reducing the number of individuals responding to stochastic samples of the value of an alternative, the group's average evaluation will become much less reliable, a phenomenon well understood by any practitioner of statistics attempting to work from limited sample sizes, and also familiar to those aware of the 'wisdom of crowds' effect in collective behaviour. Since our model is continuous it is beyond the scope of the present paper to investigate this trade-off in any detail.

References

[1] R. Bogacz, E. Brown, J. Moehlis, P. Holmes and J. D. Cohen (2006) The physics of optimal decision making: a formal analysis of models of performance in two-alternative forced-choice tasks, *Psychological Review* 113 pp. 700–765.

[2] T. D. Hanks, M. E. Mazurek, R. Kiani, E. Hopp and M. N. Shadlen (2011) Elapsed decision time affects the weighting of prior probability in a perceptual decision task, *The Journal of Neuroscience* 17 pp. 6339–6352.

[3] H. K. Khalil (1992) *Nonlinear Systems.* Macmillan, New York.

[4] T. D. Seeley, P. K. Visscher, T. Schlegel, P. M. Hogan, N. R. Franks and J. A. R. Marshall (2012) Stop signals provide cross inhibition in collective decision-making by honeybee swarms. *Science* 335 pp.108–111.
